# Awareness of Stress-Reduction Interventions on Work Attitudes: The Impact of Tenure and Staff Group in Australian Universities

**DOI:** 10.3389/fpsyg.2016.01225

**Published:** 2016-08-18

**Authors:** Silvia Pignata, Anthony H. Winefield, Chris Provis, Carolyn M. Boyd

**Affiliations:** ^1^School of Engineering, University of South AustraliaAdelaide, SA, Australia; ^2^Asia Pacific Centre for Work Health and Safety, School of Psychology, Social Work and Social Policy, University of South AustraliaAdelaide, SA, Australia; ^3^School of Management, University of South AustraliaAdelaide, SA, Australia

**Keywords:** organizational tenure, intervention awareness, universities, organizational attitudes, job satisfaction

## Abstract

This study explored the impact of staff group role and length of organizational tenure in the relationship between the awareness of stress interventions (termed intervention awareness: IA) and the work-related attitudinal outcomes of university employees. A two-wave longitudinal study of a sample of 869 employees from 13 universities completed a psychosocial work factors and health questionnaire. Hierarchical regression analyses examined the contribution of staff role and different lengths of organizational tenure with IA and employees' reports of job satisfaction, affective organizational commitment, trust in senior management, and perceived procedural justice. Employees' length of tenure affected the relation between IA and work attitudes, and there were also differences between academic and non-academic staff groups. For non-academic employees, IA predicted job satisfaction, affective organizational commitment, trust in senior management, and perceived procedural justice. However, for academics, IA only predicted job satisfaction and trust which identifies a need to increase the visibility of organizational interventions. Across the tenure groups, IA predicted: (1) perceived procedural justice for employees with five or less years of tenure; (2) job satisfaction for employees with 0–19 years of tenure; (3) trust in senior management for employees with 6–19 years of tenure; and (4) affective organizational commitment for employees with a tenure length of 6–10 years. Employees working at the university for an intermediate period had the most positive perceptions of their organization in terms of IA, job satisfaction, trust in senior management, and affective organizational commitment, whereas employees with 20–38 years of tenure had the least positive perceptions. Results suggest that employees in the middle of their careers report the most positive perceptions of their university. The findings highlight the need to attend to contextual issues in organizational stress and wellbeing interventions and suggest that management may need to implement new strategies and/or promote existing stress-management and reduction strategies to academics, and employees whom are either new to the university or those who have been working for the organization for longer periods of time to ensure that they are aware of organizational strategies to promote employee wellbeing and morale within their work environments.

## Introduction

A body of international research shows that due to economic pressures, the incidence, and severity of work-related stress in organizations is increasing (Mucci et al., 2016). In many countries, the university sector, and the working culture in universities has experienced intense change over recent decades and this has had negative impacts on the health of staff. Kinman and Court (2010) assert that workload, the pace of work, managerial, and collegial support, and levels of interpersonal conflict are of growing concern in tertiary institutions in the United Kingdom (UK). Moreover, in a review of the occupational health needs of universities in the UK, Venables and Allender (2006) indicated that academics are likely to experience particular problems with mental health. The authors found “a notably wide range of occupational hazards, and other significant factors which must be considered in planning occupational health provision for individual universities or for the sector as a whole” (p. 159). They posit that their findings in UK universities are comparable to those in other developed countries as there are broad similarities between universities in different countries. There is also research evidence that the changing and diverse work roles of academics which involve teaching, thesis supervision, research/scholarship, administration, consultancy, and community service (McInnis, 1999) are negatively impacting their wellbeing. These increasing demands have resulted in longer working hours, which have had damaging effects on the work-life balance and physical and psychological health of academics (Kinman and Jones, 2003). The international stress literature demonstrates that the situation is similar in many countries including Australia (Winefield et al., 2008), Canada (Catano et al., 2010), South Africa (Coetzee and Rothmann, 2005), the UK (Kinman and Wray, 2014), and the United States (Liu et al., 2008) showing that occupational stress has increased due to increased work demands and reduced government funding. In Canadian universities, for example, Catano et al. (2010) examined stress and its impact on health and work-related outcomes in a sample of 1440 staff from 56 universities. The authors found that with regard to strain, 13% reported high levels of psychological distress and 22% reported increased physical health symptoms. They also found that job insecurity and work-life imbalance were strong predictors of job dissatisfaction and increased levels of psychological distress.

In the context of Australian universities, a national two-wave longitudinal study of occupational stress by Winefield and colleagues found that reduced staff numbers, increased student numbers, the introduction of forced redundancies and contract appointments contributed to high levels of strain and low levels of autonomy in university staff (Winefield et al., 2008). A qualitative focus group study of work-related stress in 15 Australian universities found that university staff experienced high levels of work-related stress, with reduced funding and resources, increased workloads, fewer opportunities for career development, and reduced reward practices and recognition of staff being identified as key stressors (see Gillespie et al., 2001). The identified stressors should be considered in planning for the provision of occupational health services (Venables and Allender, 2006) and may assist university management to develop and implement stress intervention strategies. A study by Pignata and Winefield (2015) in one Australian university of the effects of the awareness of stress-reduction interventions (termed intervention awareness: IA) on employee wellbeing and work attitudes found that employees who reported IA reported higher levels of job satisfaction, affective organizational commitment, perceptions of procedural justice, and trust in senior management than those who were not aware of the interventions. Thus, it is also necessary to promote the university's stress management and wellbeing initiatives to staff.

Academics have previously reported intrinsic work role factors such as student interaction, relationships with fellow colleagues, the prestige of academic positions, autonomy, and job variety as their areas of greatest satisfaction (Kinman, 2001; Winefield et al., 2008) however, reductions in tenure, autonomy, and the collegiality inherent in academic work have affected their levels of wellbeing. In a report on the attractiveness of the academic profession in Australia, Coates et al. (2009) note that in comparison to international colleagues, Australian academics report lower levels of job satisfaction, have greater propensity for job change, and work among the longest hours per week with senior faculty working the highest of any group internationally. Venables and Allender (2006) report that a high percentage of the UK university workforce work on fixed-term teaching or research contracts which, due to their short-term commitment to the university, has implications for the way that employees work, and how they are managed. The ease of access to smart phones and other devices has allowed digital work to intrude into the non-work domain with email and other online technologies contributing towards work-life conflict and work stress. A study in one Australian university (Pignata et al., 2015) examining email volume and email management strategies found that staff associated the unnecessary use of emails by staff and students, expectations of quick responses, and high levels of email traffic with increases in work-related stress. Thus, there is a clear need for organizational initiatives to reduce stress and enhance employees' health and wellbeing.

**Figure 1:**
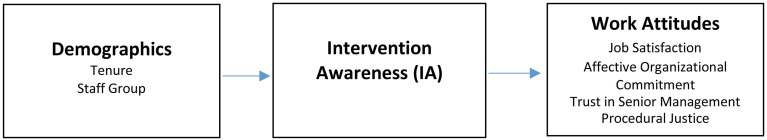
**Model for the study**.

The key aims of the present study are to examine whether employees' length of organizational tenure and/or their membership of a staff group (academic or non-academic) is related to their awareness of stress intervention strategies implemented at their university and if this has an influence on their job satisfaction, affective organizational commitment, trust in senior management, and perceptions of procedural justice (see Figure 1). Thus, the present study's focus on tenure and staff group within a longitudinal sample of university staff extends the work by Pignata et al. (2016) who examined IA and the employee level consequences on psychological strain, affective organizational commitment, and job satisfaction. As Pignata and colleagues found that the category of staff group status predicted employee levels of affective commitment to the organization, further research was undertaken to delve deeper into the two distinct occupational groups and to also examine the effect of employees' length of organizational tenure.

Research has shown that individuals differ in what they find satisfying in their jobs. Whilst non-academic employees work in diverse roles that include professional (e.g., accountant), clerical, technical, and service work, there are marked differences in the mainly administrative roles of non-academic staff and the functional roles of academics who are engaged in teaching and research. Evidence of the differences between staff populations in the university context (see Winefield et al., 2003) show that in terms of job satisfaction, 74% of non-academic staff expressed overall job satisfaction, whereas only 61% of academic staff expressed overall job satisfaction. Indeed, research by Winefield and colleagues showed that academic staff reported less affective organizational commitment, job satisfaction, and trust in senior management compared to non-academic staff (Winefield et al., 2008). Given the functional differences in staff groupings within universities, and as Pignata et al. (2016) have proposed that IA is a form of perceived organizational support which is positively associated with job satisfaction, organizational commitment, and trust in management (Rhoades and Eisenberger, 2002), we predict that in relation to IA, non-academic staff will report more positive associations with job satisfaction, affective commitment to the organization, and trust in senior management than academics *(Hypothesis 1)*.

Stress and psychological risk at work can be conceptualized as an imbalance of job demands and job resources. For example, the theoretical framework of the Job Demands-Resources model (Demerouti et al., 2001) proposes that high job demands (i.e., work overload, interpersonal conflict, job insecurity) lead to strain and health impairment (via the health impairment process), whereas high resources (i.e., feedback, job control, social support) lead to improved motivation, and productivity (via the motivational process; Schaufeli and Taris, 2014). Thus, in psychologically healthy workplaces, work demands, and available job resources are in balance. Conversely, in unhealthy workplaces, high job demands may lead to exhaustion, whilst low job resources may lead to disengagement which are both symptoms of burnout. In an examination of the management of academics in terms of balancing job demands and job resources, Barkhuizen et al. (2014) showed that academics experienced high job demands compared to the availability of job resources. Given this imbalance in demands and resources, and on the basis of the social-exchange reciprocity norm that suggests that academics may perceive this imbalance in the organization's processes of allocating resources as unjust, we propose that in relation to IA, non-academic staff will report more positive associations with perceived procedural justice than academics *(Hypothesis 2)*.

The present study also investigates length of organizational tenure in relation to work-related outcomes such as job satisfaction as they may be explained by Herzberg's two-factor theory (see Herzberg et al., 1957) which considers job satisfaction (a function of motivating factors e.g., achievement, advancement) and dissatisfaction (a function of hygiene factors e.g., supervision, company policy, and administration) as distinct constructs. The model posits that workers in the early stages of their careers usually experienced low job satisfaction due to unfulfilled work expectations. However, when those workers advanced in their careers they gained maturity and work experience which in turn, led to higher levels of job satisfaction. It is clear that gaining experience at work is an outcome of tenure, and that work experience is a requirement for promotion opportunities and the inherent reward and salary benefits. Work experience may also lead to a sense of mastery, which would be related to the motivating factors inherent in job satisfaction. Furthermore, research has shown that employees who have been with the organization for longer periods may have developed a favorable view of their treatment by the organization (Rhoades and Eisenberger, 2002) and this may be associated with increased morale and wellbeing outcomes. Indeed, the long-term development of employee wellbeing was examined by Mäkikangas et al. (2015) over a 10-year period and the development of favorable affective wellbeing was found to be eight times more probable than the development of unfavorable affective wellbeing. In a systematic review of the findings of 40 studies on the long-term development of affective employee wellbeing (accounting for the effects of time lag, age, and job change Mäkikangas et al., 2016) found that age and change of job were the major factors influencing stability, with younger employees and job changers tending to display greater across-time changes in wellbeing than older employees and those who have stayed in the job. As Bentley et al. (2013) report that levels of job satisfaction are higher amongst recently promoted academics and lower amongst mid-career academics, we propose that longer tenured employees will have greater maturity, experience, and mastery of their job and will perceive that their organization supports them by offering stress intervention strategies. Thus, we predict that longer lengths of organizational tenure will positively affect the relationship between IA and employees' levels of job satisfaction *(Hypothesis 3)*.

As trustworthiness, perceived procedural justice, and affective commitment to the organization are attitudes that develop over time and can be enhanced by positive experiences (Arnold et al., 1995; Holtz and Harold, 2009) they highlight the reciprocal social-exchange process between employee and their employer and suggest that when employees feel valued and supported by their employing organization, they may perceive senior management as being trustworthy, or perceive that organizational policies and practices are just (Pignata et al., 2016). As longer term employees are likely to have had more opportunities for positive exchanges with their organization, and as assessments of organizational procedures (in terms of procedural justice) can influence employee's levels of trust (Folger and Konovsky, 1989; Saunders and Thornhill, 2003), we predict that there will be a positive association with organizational tenure in that longer lengths of organizational tenure will positively affect the relationship between IA and employees' level of trust in senior management, procedural justice, and affective organizational commitment *(Hypothesis 4)*.

## Method

### Participants and procedure

Of the 969 participants who responded to both the 2000 (Time 1:T1) and 2003/4 (Time 2:T2) waves of a study of occupational stress at a representative sample of 13 Australian public universities (see Winefield et al., 2008 for characteristics of the universities), the present study focused on the 869 participants who remained employed at the same university for the period of the two-wave study and responded to the measure of IA. Like the sample examined in Pignata et al. (2016), this sample of 869 salaried staff (casual or hourly paid staff did not participate in the study) comprised 327 men (38%) and 542 women (62%). The average age in 2003 was 46.5 years (*SD* = 9.24) while the average length of tenure was 12.1 years (*SD* = 7.34). The composition of the sample was 372 (43%) academic and 497 (57%) non-academic staff which is broadly representative of the 2003 national profile of 42% academic and 58% non-academic staff (Department of Education Science and Training, 2003). The majority of the sample were non-academic staff who worked in clerical / administrative (*n* = 237, 27%), non-academic (*n* = 147, 17%), technical (*n* = 91, 10%), general service (*n* = 7, 1%), or other classifications (*n* = 7, 1%). The sample of academics comprised tutors/ research assistants (*n* = 61, 7%), lecturers (*n* = 124, 14%), senior lecturers (*n* = 114, 13%), Associate Professors /Professors (*n* = 61, 7%), and other classifications (e.g., Deans; *n* = 7, 1%). No job classification was reported by 13 (2%) respondents.

Participation in both survey waves was anonymous but data were matched across surveys using code identifiers as staff were asked to provide the first three letters of their mother's maiden name (if unknown, the first three letters of their mother's first name), the first three letters of their father's first name, and their date of birth and gender. Thirteen of the 17 universities that participated in the first survey took part in the second. The surveys were completed by 6756 participants at T1, giving a response rate of 25%. At T2, the surveys were completed by 6321 participants, a response rate of 26%. The longitudinal sample of 869 participants represent approximately 13% of the T1 respondents. In contrast to the use of a paper survey at T1, the T2 survey was administered electronically and was conducted between October and December 2003 at 12 of the universities, and 1 year later at the remaining university. It should be noted that the measurement equivalence of paper-and-pencil and organizational internet surveys was demonstrated in a large-scale examination by De Beuckelaer and Lievens (2009). The longitudinal study received ethics approval from the University of South Australia's Human Research and Ethics Committee. All non-casual staff at each university were emailed an invitation letter providing details of the nature and purpose of the survey and how to access the survey website. Reminder notices were emailed to staff 2, 4, and 6 weeks thereafter.

### Measures

Both the T1 and T2 survey questionnaires sought demographic details (i.e., age, gender). The following work attitude measures were used in the survey, and each had internal reliabilities of between 0.69 and 0.96 (Cronbach's alpha coefficients).

The mean for job satisfaction is based on the total scores, whereas the measures of affective organizational commitment, perceived procedural justice and trust in senior management are based on the item means. It should be noted that the following five measures (job satisfaction, affective organizational commitment, trust in senior management, procedural justice, and IA) were also employed in the study by Pignata et al. (2016).

#### Job satisfaction

The 15-item scale developed by Warr et al. (1979) measured job satisfaction. The scale assessed the intrinsic (i.e., “How satisfied or dissatisfied do you feel with your opportunity to use your abilities?”) and the extrinsic aspect of job satisfaction, for example, “How satisfied or dissatisfied do you feel with your job security? Each item was scored on a 7-point Likert scale (1 = *extremely dissatisfied*; 7 = *extremely satisfied*).

#### Affective organizational commitment

Five items from Porter et al.'s (1974) scale measured affective organizational commitment. An example item is “I really care about the future of this university.” Each item was scored on a 5-point scale (1 = *strongly disagree*; 5 = *strongly agree*).

#### Trust in senior management

An 8-item scale developed from Mayer and Davis (1999) and Butler (1991) measured trust in senior management (e.g., “Senior Management of my University treat staff fairly”). Each item was scored on a 5-point scale (1 = *strongly disagree*; 5 = *strongly agree*).

#### Procedural justice

A 4-item scale developed by Gillespie et al. (2001) measured perceptions of procedural justice (e.g., “Staff performance is fairly appraised”). Each item was scored on a 5-point scale (1 = *strongly disagree*; 5 = *strongly agree*).

#### IA

We used an intervention evaluation component in the T2 survey in which university staff were asked: “During the past 3 years has your university undertaken any measures to reduce stress among its employees?” Response options were (1 = *Yes*, 2 = *No*, 3 = *Don't Know*). These options were then coded in a binary format (0 = *No/Don't Know*, 1 = *Yes*) to distinguish between those with a positive awareness of stress-reduction measures and those with negative or neutral perceptions.

#### Length of organizational tenure

Respondents were asked to complete the number of years that they had worked at the university. The responses were recoded into four tenure groups (0–5 years, 6–10 years, 11–19 years, 20–38 years).

#### Staff group

Respondents were asked to state whether they were a member of academic or non-academic staff (1 = *academic*, 2 = *non-academic*).

#### Control variables

To reduce the possibility of spurious relationships introduced by demographic characteristics, two control variables were entered in all the equations: age, and gender (1 = *male*, 2 = *female*).

### Analyses

Hierarchical regression analyses were performed using SPSS Statistics 17.0 software. Preliminary checks were conducted to ensure that there was no violation of the assumptions of multicollinearity, normality, linearity, and homoscedasticity. Two multivariate outliers were identified through Mahalanobis distance with *p* < 0.001, and were deleted. Listwise deletion of missing data was used in all analyses. As prior levels of the dependent variable (i.e., T1 levels of job satisfaction, affective organizational commitment, trust in senior management, perceived procedural justice) were included as additional predictors in the analysis, tests of the abovementioned relationships are rigorous as they show that the predictors account for changes over time in the levels of the dependent variables (Zapf et al., 1996).

## Results

The means, standard deviations, internal reliability coefficients (Cronbach's alpha), and bivariate correlations are displayed in Table 1. Although none of the bivariate correlations was high enough to suggest that any of the self-report measures were assessing the same constructs (see Nunnally and Bernstein, 1994), job satisfaction at T2 was strongly associated with the T2 variables of perceived procedural justice (*r* = 0.67) and trust in senior management (*r* = 0.57). Affective organizational commitment at T2 was also strongly associated with the T2 variable of trust in senior management (*r* = 0.56).

**Table 1:** **Descriptive statistics and variable inter-correlations**.

**Variable**		**M^a^**	**SD**	**α**	**1**	**2**	**3**	**4**	**5**	**6**	**7**	**8**	**9**	**10**	**11**	**12**
1.	Age	46.50	9.24	—												
2.	Gender	—	—	—	−0.15^**^											
3.	Tenure	12.08	7.34	—	0.46^**^	−0.09^*^										
4.	Group	—	—	—	−0.19^**^	0.20^**^	−0.07									
5.	JS T1	66.26	13.39	0.87	−0.06	0.13^**^	−0.13^**^	0.18^**^								
6.	JS T2	65.43	14.89	0.89	−0.03	0.11^**^	−0.04	0.17^**^	0.63^**^							
7.	AC T1	3.49	0.69	0.79	−0.01	0.08^*^	−0.09^**^	0.18^**^	0.49^**^	0.34^**^						
8.	AC T2	3.51	0.79	0.84	−0.04	0.10^**^	−0.03	0.19^**^	0.41^**^	0.53^**^	0.62^**^					
9.	TM T1	2.53	0.86	0.95	−0.08^*^	0.03	−0.18^**^	0.19^**^	0.55^**^	0.40^**^	0.45^**^	0.40^**^				
10.	TM T2	2.64	0.93	0.96	−0.12^**^	0.05	−0.14^**^	0.18^**^	0.44^**^	0.57^**^	0.38^**^	0.56^**^	0.59^**^			
11.	PJ T1	3.06	0.67	0.69	0.00	0.03	−0.07^*^	−0.02	0.54^**^	0.42^**^	0.31^**^	0.29^**^	0.41^**^	0.36^**^		
12.	PJ T2	3.09	0.82	0.76	−0.01	−0.01	0.03	0.05	0.42^**^	0.67^**^	0.23^**^	0.41^**^	0.33^**^	0.51^**^	0.52^**^	
13.	IA	0.21	0.41	—	−0.03	0.06	−0.04	0.18^**^	0.15^**^	0.24^**^	0.15^**^	0.18^**^	0.16^**^	0.21^**^	0.05	0.15^**^

*N = 681−869. JS, Job satisfaction; AC, Affective organizational commitment; TM, Trust in senior management; PJ, Procedural justice, IA, Intervention awareness*.

a *For JS, item scores were summed. Item scores were averaged for AC, TM, and PJ*.

* *p < 0.05*.

** *p < 0.01*.

^***^p < 0.001.

### Analysis of interaction effects

Analyses assessed the contribution of staff group and tenure on IA controlling for age, gender, tenure, staff group, the interaction between length of organizational tenure, and staff group, and T1 variable differences. Thus, age, gender, tenure, and staff group were entered into the analyses. In addition, to examine possible interactions between tenure and staff group, a product term was calculated in accordance with the method suggested by Cohen and Cohen (1983) and Jaccard et al. (1990). This method involved three steps. First, tenure scores were “centered” (i.e., mean = 0) to reduce the problem of multicollinearity in the subsequent analysis. Secondly, the centered tenure scores were multiplied by the dummy variable scores for staff group to obtain a product term. Third, this product term was entered into the regression analysis following tenure and staff group. A significant interaction was deemed to have occurred if the addition of this term produced (or resulted in) a significant increase in *R*^2^.

The regression tables display the unstandardized regression coefficients (B) and their standard errors, the standardized regression coefficients, (β) and adjusted *R*^2^ for each step.

As can be observed in Table 2, the T1 levels of the attitudinal outcome variables were predictors of the T2 outcomes. Staff group remained a predictor of affective organizational commitment after the addition of IA in Step 2, whereas staff group was only a significant predictor of trust in senior management at Step 1. With regard to perceived procedural justice, it should be noted that staff group and the interaction between staff group and tenure, predicted procedural justice prior to the addition of IA. In view of these findings, further regression analyses were undertaken to examine differences between academic and non-academic staff in the effect of IA on affective organizational commitment, and other work attitudinal outcomes.

**Table 2:** **Hierarchical regression analyses for relations between IA and T2 work attitudes controlling for age, gender, tenure, staff group, the interaction between tenure and staff group, and T1 variables**.

	**Step 1**					**Step 2**				
**Measure**	***B***	***SE B***	**β**	***t***		***B***	***SE B***	**β**	***t***	
**JOB SATISFACTION**										
Age	0.02	0.05	0.02	0.46		0.02	0.05	0.01	0.30	
Gender	0.83	0.85	0.03	0.97		0.79	0.84	0.03	0.95	
Tenure	−0.06	0.18	−0.03	−0.30		−0.04	0.18	−0.02	−0.24	
Staff Group	1.58	0.84	0.05	1.87		0.93	0.84	0.03	1.11	
Tenure × Staff Group	0.11	0.11	0.08	0.96		0.10	0.11	0.08	0.95	
T1 Satisfaction	0.68	0.03	0.62	22.15^***^		0.66	0.03	0.60	21.66^***^	
IA						4.50	1.00	0.14	5.00^***^	
*Adj. R*^2^					0.40					
*F*					91.03^***^					
Δ*R*^2^										0.02
*F* change										25.04^***^
**AFFECTIVE ORGANIZATIONAL COMMITMENT**										
Age	−0.02	0.01	−0.04	−1.27		−0.02	0.01	−0.04	−1.32	
Gender	0.28	0.22	0.04	1.27		0.27	0.22	0.03	1.23	
Tenure	−0.03	0.05	−0.05	−0.55		−0.02	0.05	−0.05	−0.52	
Staff Group	0.59	0.22	0.07	2.67^**^		0.49	0.22	0.06	2.19^*^	
Tenure × Staff Group	0.03	0.03	0.10	1.19		0.03	0.03	0.10	1.20	
T1 Commitment	0.70	0.03	0.61	22.49^***^		0.68	0.03	0.60	22.10^***^	
IA						0.78	0.26	0.08	2.98^**^	
*Adj. R*^2^					0.40					
*F*					95.36^***^					
Δ*R*^2^										0.01
*F* change										8.85^**^
**TRUST IN SENIOR MANAGEMENT**										
Age	−0.05	0.03	−0.06	−1.71		−0.05	0.03	−0.06	−1.84	
Gender	0.22	0.44	0.01	0.50		0.16	0.44	0.01	0.37	
Tenure	−0.12	0.09	−0.11	−1.25		−0.11	0.09	−0.11	−1.18	
Staff Group	0.90	0.44	0.06	2.03^*^		0.61	0.44	0.04	1.38	
Tenure × Staff Group	0.08	0.06	0.12	1.34		0.07	0.06	0.12	1.29	
T1 Trust	0.63	0.03	0.57	20.01^***^		0.61	0.03	0.56	19.58^***^	
IA						2.09	0.52	0.11	4.02^***^	
*Adj. R*^2^					0.35					
*F*					77.55^***^					
Δ*R*^2^										0.01
*F* change										16.12^***^
**PROCEDURAL JUSTICE**										
Age	−0.01	0.01	−0.03	−0.86		−0.01	0.01	−0.03	−0.86	
Gender	−0.24	0.23	−0.04	−1.04		−0.26	0.23	−0.04	−1.13	
Tenure	−0.06	0.05	−0.14	−1.31		−0.06	0.05	−0.13	−1.18	
Staff Group	0.48	0.22	0.07	2.16^*^		0.38	0.23	0.06	1.68	
Tenure × Staff Group	0.06	0.03	0.21	1.99^*^		0.06	0.03	0.20	1.91	
T1 Justice	0.63	0.04	0.53	16.24^***^		0.63	0.04	0.53	16.12^***^	
IA						0.84	0.27	0.10	3.13^**^	
*Adj. R*^2^					0.28					
*F*					45.19^***^					
Δ*R*^2^										0.01
*F* change										9.77^**^

*N = 673–869. ΔR^2^, change in R^2^. IA, Intervention awareness*.

* *p ≤ 0.05*.

** *p ≤ 0.01*.

*** *p ≤ 0.001*.

### Analyses examining staff group differences

Hierarchical regression analyses examined differences between academic and non-academic staff in terms of the impact of IA on job satisfaction, affective organizational commitment, trust in senior management, and perceived procedural justice. In all cases, IA was the independent variable with two levels (*No/Don't Know*, and *Yes*). Respondents' age, gender, tenure, and their scores on the relevant T1 variables were included in the analyses as control variables at Step 1.

As shown in Table 3, the T1 level variables of job satisfaction, affective organizational commitment, trust in senior management, and perceived procedural justice were predictors of job satisfaction, affective organizational commitment, trust, and justice, respectively, for academic staff. In addition, tenure was a predictor of affective organizational commitment at Step 1 only. When IA was added to the analyses at Step 2, its effect was significant for job satisfaction, and trust in senior management, but not for affective organizational commitment and perceived procedural justice.

**Table 3:** **Relationships between IA and T2 work attitudes: academic staff**.

	**Step 1**					**Step 2**				
**Measure**	***B***	***SE B***	**β**	***t***		***B***	***SE B***	**β**	***t***	
**JOB SATISFACTION**										
Age	0.00	0.08	0.00	0.04		−0.01	0.08	−0.01	−0.10	
Gender	0.20	1.11	0.01	0.18		0.16	1.10	0.01	0.15	
Tenure	0.07	0.09	0.04	0.83		0.09	0.09	0.05	0.97	
T1 Satisfaction	0.72	0.04	0.69	17.16^***^		0.71	0.04	0.68	16.71^***^	
IA						4.02	1.68	0.09	2.40^*^	
*Adj. R*^2^					0.46					
*F*					76.34^***^					
Δ*R*^2^										0.01
*F* change										5.71^*^
**AFFECTIVE ORGANIZATIONAL COMMITMENT**										
Age	−0.04	0.02	−0.08	−1.71		−0.04	0.02	−0.09	−1.74	
Gender	0.17	0.34	0.02	0.50		0.17	0.38	0.02	0.50	
Tenure	0.02	0.03	0.04	0.85^*^		0.02	0.03	0.05	0.91	
T1 Commitment	0.70	0.05	0.62	14.95^***^		0.69	0.05	0.61	14.70^***^	
IA						0.92	0.52	0.07	1.78	
*Adj. R*^2^					0.38					
*F*					58.12^***^					
Δ*R*^2^										0.01
*F* change										3.16
**TRUST IN SENIOR MANAGEMENT**										
Age	−0.06	0.04	−0.06	−1.28		−0.06	0.04	−0.07	−1.37	
Gender	−0.00	0.62	0.00	−0.00		−0.03	0.062	−0.00	−0.05	
Tenure	−0.04	0.05	−0.04	−0.74		−0.03	0.05	−0.03	−0.66	
T1 Trust	0.62	0.04	0.60	14.02^***^		0.60	0.05	0.57	13.31^***^	
IA						2.56	0.97	0.11	2.66^**^	
*Adj. R*^2^					0.37					
*F*					54.08^***^					
Δ*R*^2^										0.01
*F* change										7.06^**^
**PROCEDURAL JUSTICE**										
Age	−0.01	0.02	−0.02	−0.39		−0.01	0.02	−0.02	−0.38	
Gender	−0.42	0.32	−0.06	−1.30		−0.42	0.32	−0.06	−1.31	
Tenure	−0.01	0.03	−0.02	−0.27		−0.00	0.03	−0.01	−0.17	
T1 Justice	0.72	0.06	0.59	12.23^***^		0.72	0.06	0.58	12.18^***^	
IA						0.53	0.48	0.05	1.10	
*Adj. R*^2^					0.33					
*F*					37.62^***^					
Δ*R*^2^										0.01
*F* change										1.22

*N = 294–372. ΔR^2^, change in R^2^. IA, Intervention awareness*.

* *p ≤ 0.05*.

** *p ≤ 0.01*.

*** *p ≤ 0.001*.

For non-academic staff, Table 4 shows that the T1 levels of job satisfaction, affective commitment to the organization, trust and procedural justice were predictors of the T2 levels of job satisfaction, affective organizational commitment, trust in senior management, and procedural justice, respectively. Furthermore, tenure remained a predictor of procedural justice for non-academic staff. However, unlike the results for academic staff, the effect of IA was significant for all four attitudinal outcomes.

**Table 4:** **Relationships between IA and T2 work attitudes: non-academic staff**.

	**Step 1**					**Step 2**				
**Measure**	***B***	***SE B***	**β**	***t***		***B***	***SE B***	**β**	***t***	
**JOB SATISFACTION**										
Age	0.04	0.07	0.03	0.59		0.03	0.07	0.02	0.50	
Gender	1.45	1.26	0.04	1.15		1.43	1.24	0.04	1.15	
Tenure	0.16	0.09	0.07	1.78		0.16	0.09	0.07	1.78	
T1 Satisfaction	0.63	0.04	0.55	14.55^***^		0.63	0.04	0.55	14.55^***^	
IA						5.41	1.26	0.16	4.28^***^	
*Adj. R*^2^					0.33					
*F*					58.23^***^					
Δ*R*^2^										0.03
*F* change										18.30^***^
**AFFECTIVE ORGANIZATIONAL COMMITMENT**										
Age	−0.00	0.02	−0.01	−0.27		−0.01	0.02	−0.01	−0.31	
Gender	0.40	0.29	0.05	1.35		0.38	0.29	0.05	1.29	
Tenure	0.04	0.02	0.07	1.77		0.04	0.02	0.07	1.83	
T1 Commitment	0.69	0.04	0.60	16.74^***^		0.68	0.04	0.59	16.44^***^	
IA						0.72	0.30	0.09	2.42^*^	
*Adj. R*^2^					0.36					
*F*					71.27^***^					
Δ*R*^2^										0.01
*F* change										5.86^***^
**TRUST IN SENIOR MANAGEMENT**										
Age	−0.04	0.03	−0.05	−1.15		−0.04	0.03	−0.05	−1.25	
Gender	0.43	0.62	0.03	0.69		0.35	0.62	0.02	0.57	
Tenure	0.03	0.04	0.03	0.77		0.03	0.04	0.03	0.79	
T1 Trust	0.63	0.04	0.56	14.31^***^		0.62	0.04	0.55	14.18^***^	
IA						1.91	0.63	0.12	3.05^**^	
*Adj. R*^2^					0.30					
*F*					52.44^***^					
Δ*R*^2^										0.01
*F* change										9.32^**^
**PROCEDURAL JUSTICE**										
Age	−0.01	0.02	−0.03	−0.63		−0.01	0.02	−0.03	−0.64	
Gender	−0.11	0.32	−0.02	−0.34		−0.15	0.32	−0.02	−0.48	
Tenure	0.05	0.02	0.12	2.37^*^		0.05	0.02	0.12	2.46^*^	
T1 Justice	0.57	0.05	0.49	10.99^***^		0.56	0.05	0.48	10.83^***^	
IA						1.00	0.33	0.14	3.04^**^	
*Adj. R*^2^					0.24					
*F*					31.07^***^					
Δ*R*^2^										0.02
*F* change										9.24^**^

*N = 379–497. ΔR^2^, change in R^2^. IA, Intervention awareness*.

* *p ≤ 0.05*.

** *p ≤ 0.01*.

*** *p ≤ 0.001*.

### Analyses examining different lengths of tenure

Hierarchical regression analyses examined differing lengths of tenure in the inter-relationships between IA and employee levels of job satisfaction, affective organizational commitment, trust in senior management, and perceptions of procedural justice. In all cases, IA was the independent variable with two levels (*No/Don't Know*, and *Yes*). Age, gender, staff group, and respondents' scores on the relevant T1 variables were included in analyses as control variables at Step 1.

Respondents reported the number of years that they had worked at the university and their responses were recoded into four tenure groups (0–5 years, 6–10 years, 11–19 years, and 20–38 years). Each of these samples met the sample size criteria recommended by Tabachnick and Fidell (2001, p. 117) for multiple regression analyses. According to Tabachnick and Fidell's formula (*N* > 50 + 8 m where *m* = the number of independent variables), and taking into account the five independent variables used in the analyses, 90 cases were required for a reliable equation.

Table 5 shows the relation of IA to work attitudes for each of the four tenure groups. For university staff with 5 or fewer years of organizational tenure, there were significant positive relationships (albeit small) between IA and job satisfaction and perceived procedural justice when prior levels of job satisfaction and justice, respectively, were included as additional predictors in the hierarchical regression analyses. For all attitudinal outcomes, the T1 level of the dependent variable was a predictor of the T2 attitudinal outcome.

**Table 5:** **Relation of IA to T2 work attitudes by length of tenure**.

**Work Attitude**		**Length of Tenure**							
		**0–5 years **(*****n***** = **120–175)**		**6–10 years **(*****n***** = **199–254)**		**11–19 years **(*****n***** = **241–306)**		**20–38 years **(*****n***** = **113–134)**	
**Step**	**Predictors**	**β**	**Δ*R*^2^**	**β**	**Δ*R*^2^**	**β**	**Δ*R*^2^**	**β**	**Δ*R*^2^**
**JOB SATISFACTION**									
1	Age	0.02	0.19^***^	−0.00	0.42^***^	0.04	0.44^***^	0.04	0.54^***^
	Gender	0.06		0.06		−0.01		−0.05	
	Staff Group	−0.10		0.12^*^		0.07		0.07	
	T1 Satisfaction	0.44^***^		0.62^***^		0.65^***^		0.73^***^	
2	IA	0.16^*^	0.03^*^	0.17^**^	0.03^**^	0.12^**^	0.01^**^	0.05	0.00
	Total *R*^2^		0.22^***^		0.45^***^		.45^***^		0.54^***^
**AFFECTIVE ORGANIZATIONAL COMMITMENT**									
1	Age	−0.01	0.26^***^	−0.03	0.34^***^	−0.05	0.45^***^	−0.08	0.55^***^
	Gender	−0.07		0.10		0.03		0.09	
	Staff Group	−0.05		0.17^**^	0.14^**^	0.05		0.06	
	T1 Commitment	0.53^***^		0.51^***^		0.66^***^		0.70^***^	
2	IA	0.06	0.00	0.14^**^	0.02^**^	0.05	0.00	0.02	0.00
	Total *R*^2^		0.26^***^		0.36^***^		0.45^***^		0.55^***^
**TRUST IN SENIOR MANAGEMENT**									
1	Age	−0.04	0.25^***^	−0.08	0.35^***^	−0.04	0.34^***^	−0.09	0.43^***^
	Gender	−0.04		−0.02		0.05		0.05	
	Staff Group	−0.03		0.11		0.04		0.11	
	T1 Trust	0.51^***^		0.56^***^		0.58^***^		0.62^***^	.
2	IA	0.06	0.00	0.24^***^	0.05^***^	0.12^*^	0.01^*^	−0.05	0.00
	Total *R*^2^		0.25^***^		0.40^***^		0.35^***^		0.43^***^
**PROCEDURAL JUSTICE**									
1	Age	−0.01	0.16^***^	−0.10	0.25^***^	−0.00	0.28^***^	−0.01	0.46^***^
	Gender	−0.12		0.01		−0.08		0.02	
	Staff Group	−0.12		0.13		0.09		0.14	
	T1 Justice	0.41^***^		0.50^***^		0.54^***^		0.67^***^	
2	IA	0.17^*^	0.03^*^	0.06	0.00	0.10	0.01	0.07	0.01
	Total *R*^2^		0.19^***^		0.25^***^		0.29^***^		0.47^***^

*ΔR^2^ = R^2^ change*.

* *p ≤ 0.05*.

** *p ≤ 0.01*.

*** *p ≤ 0.001*.

For employees with 6–10 years length of tenure, IA was a predictor of higher levels of job satisfaction, affective commitment to the organization and trust in senior management when prior levels of those respective variables were included as additional predictors in the analyses. For all attitudinal outcomes, the T1 level of the dependent variable was a predictor of the T2 outcome. Of particular note, staff group (i.e., being a member of non-academic staff) predicted affective commitment to the organization and remained a predictor of higher levels of commitment when both IA and prior levels of commitment were included as additional predictors in the analyses.

For employees with 11–19 years of tenure, IA predicted higher levels of job satisfaction and perceptions of trust in senior management when prior levels of those respective variables were included as additional predictors in the analyses. For all work-attitude outcomes, the T1 level of the dependent variable predicted the T2 outcome.

With regard to university employees with 20–38 years of organizational tenure, there were no relationships between the awareness of stress-reduction interventions, and job satisfaction, commitment to the organization, trust in senior management, or perceived procedural justice. However, T1 levels of the dependent variables predicted their corresponding T2 attitudinal outcomes.

## Discussion

The present study examined the inter-relationships between staff group role, length of organizational tenure, IA (in the positive awareness of intervention strategies), and job satisfaction and other positive work attitudinal outcomes. First, the study looked at academic and non-academic staff separately to examine the contribution of IA on the attitudinal outcomes controlling for age, gender, and T1 variable differences. With regard to hypothesis 1, the study found that for academics, IA predicted both job satisfaction and trust in senior management. The results for non-academic staff were more positive as IA predicted job satisfaction, affective organizational commitment, and trust in senior management supporting hypothesis 1. Thus, in terms of job satisfaction for both staff groups, IA may be considered an aspect of perceived organizational support (POS) as this finding is in line with the literature on social-exchange theory, as a body of job satisfaction research including a meta-analytic review by Riggle et al. (2009) found that POS has a strong, positive effect on job satisfaction. Indeed, Rhoades and Eisenberger (2002, p. 701) propose that “POS should contribute to overall job satisfaction by meeting socio-emotional needs, increasing performance-reward expectancies, and signaling the availability of aid when needed.” Furthermore, as trust evolves over time through the repeated interactions of values and attitudes within the exchange relationship between employee and organization (Jones and George, 1998), this result is consistent with research by Whitener (2001) that POS was related to trust in management. Given that there was no association between IA and affective organizational commitment for the academic group, this suggests that organizational grouping impacts on employees' emotional attachment to their organization. This may require university management to build the cohesiveness of employees within the organization as research has demonstrated the organizational benefits of a strongly committed workforce in terms of enhancing job performance and organizational citizenship behaviors (Meyer et al., 2002; Riketta, 2002). The finding is also in line with previous research that has indicated that academics reported more adverse work experiences than non-academic respondents (Winefield et al., 2008) so there is a clear need to focus on the talent management of academics to enhance the mutual exchange processes between them and their organization.

The second hypothesis that non-academic staff would report more positive associations with IA and perceived procedural justice than academics, was also supported as there was no effect of IA on justice for academic staff whereas for non-academic staff, IA predicted perceived procedural justice. In addition, tenure also predicted procedural justice which is not surprising given that the social exchange processes between employee-employer are shaped over time. There was partial support for hypothesis 3 that longer lengths of organizational tenure would positively affect the relationship between IA and employees' levels of job satisfaction. However, this assertion only applied for employees with up to 19 years of tenure and not those holding 20–38 years of tenure. The adverse result for longer tenured employees is of particular interest and requires further investigation as research has shown that positive work experiences enhance employees' perceptions of trust, justice and commitment (Arnold et al., 1995; Holtz and Harold, 2009) and that the perception of fair treatment is critical to the continuation of relational psychological contracts (Rousseau and Parks, 1992).

There was partial support for the fourth hypothesis that longer lengths of organizational tenure will positively affect the relationship between IA and employees' level of trust in senior management, procedural justice, and affective organizational commitment. However, this was only for staff with tenure lengths of 6–10 years for the outcomes of affective organizational commitment and 6–19 years tenure for trust in senior management. With regard to university staff with five or less years of tenure, IA only predicted higher levels of perceptions of procedural justice even when prior levels of justice was included as additional predictors in the hierarchical regression analyses. For employees with 6–10 years of tenure, IA predicted higher levels of affective organizational commitment and perceptions of trust in senior management. Of particular note, being a member of non-academic staff with between 6–10 years of tenure predicted affective commitment to the organization and remained a predictor of higher levels of commitment when both IA and prior levels of commitment were included as additional predictors. With regard to employees with 11–19 years of tenure, IA only predicted higher levels of trust in senior management. However, for employees with 20–38 years of organizational tenure, there were no relationships between IA and affective commitment to the organization, trust in senior management, or perceptions of procedural justice.

In sum, employees' length of organizational tenure did affect the relation between IA and some attitudinal outcomes. Across the tenure groups, IA predicted: (1) job satisfaction for employees with 0–19 years of tenure; (2) trust in senior management for employees with 6–19 years of tenure; (3) affective organizational commitment for employees with tenure for 6–10 years; and (4) perceived procedural justice for employees with five or less years of tenure. These results suggest that employees working at the university for an intermediate period of time such as 6–10 years had the most positive perceptions of their organization in terms of the awareness of stress-reduction interventions, job satisfaction, trust, and affective commitment, whereas employees with 20–38 years of tenure had the least positive perceptions which may reflect the need to better manage and communicate organizational change initiatives to longstanding employees. Nonetheless, this finding warrants further investigation as it is intuitively appealing that a sense of mastery at work is related to having seniority and/or a longer length of organizational tenure which, in turn, is associated with greater job satisfaction and other positive work attitudes. Thus, it would be valuable, particularly in terms of employee wellbeing and supporting effective performance, to determine what factors are causing longer term employees to develop unfavorable perceptions.

Research by Winefield et al. (2008), for example, has shown that in the Australian university context, workplace factors (i.e., trust in senior management, perceived procedural justice, autonomy) were the best predictors of academics' affective commitment to their university as they accounted for 21% of the variance, whereas demographic factors (i.e., occupational level) and individual difference variables (i.e., job involvement, extraversion, negative affectivity, hardiness) accounted only for 3% and 14% of the variance, respectively. On the other hand, for non-academic staff, the individual difference variables of job involvement, extraversion, conscientiousness, hardiness, and maladaptive coping were more predictive of affective commitment than the workplace factors of trust, justice, and autonomy (Winefield et al., 2008). These results suggest that the roles played by perceived work conditions and individual variables in predicting commitment differ between academic and non-academic employees. As a result, Human Resource practices (i.e., reward, promotion, incentive practices) and interventions may need to be tailored to take account of these group differences. Future research should also focus more on trust in management (both senior and line managers) and perceptions of procedural justice as key mechanisms to develop affective organizational commitment and job satisfaction during the professional career.

### Limitations

The results suggest that the positive perception of stress-reduction intervention strategies implemented within university workplaces appear to enhance employee job satisfaction, trust in senior management, affective commitment to the organization and perceptions of procedural justice for some staff groups. The results of this study emphasize the importance of using a longitudinal design and multiple regression analyses that incorporate the T1 levels of the dependent variables in order to examine the links from demographic characteristics such as staff group and length of tenure, and organizational resources such as IA, to work-related attitudes. Although the effect sizes for the aforementioned relationships were small, it is reasonable to assume that the positive effects of IA on these attitudes were statistically reliable. Whilst causation is not conclusive, these findings suggest a link that warrants further investigation. It is important to acknowledge the limitation of common method bias of the measurement method as only self-report questionnaire data on health and psychosocial factors were collected from the same participants in both waves of the study.

There is a need for future research in this specific area to take steps to control for the number of stress interventions implemented prior to the study period. The authors were not able to collect data regarding the number of stress-reduction initiatives implemented at each of the thirteen universities prior to the study. However, a forthcoming study by Pignata and colleagues will report on the data collected from management at five of the 13 universities that details the stress interventions implemented between the two waves of the longitudinal study.

### Implications and conclusion

The present study identifies the potential target groups for the promotion of organizational stress-reduction strategies and thus, adds to the stress intervention literature in two ways. First, it compared academic and non-academic university staff to explore potential occupation-specific associations between IA, tenure, and work-related outcomes; and secondly, it compared groups with differing lengths of tenure to examine specific associations between IA and work-related attitudes. As no previous research has examined those relationships the study fills a gap in the stress intervention literature. By examining the two distinct staff groups separately, as well as investigating employees in differing stages of their careers, the study's results have practical implications for organizations and management, particularly university management as they identify potential target groups or areas for the promotion of stress-reduction strategies. Management at each of the individual universities may benefit from conducting independent assessments in order to identify specific areas that require attention. Given the need to attend to contextual and process issues in organizational stress and wellbeing interventions (Biron and Karanika-Murray, 2014), the findings suggest that university management may need to implement new strategies and promote existing stress-management and reduction strategies to academic staff, and employees whom are either new to the university or those who have been working for the organization for longer periods of time, to ensure that those employees are aware of organizational strategies to promote and enhance employee wellbeing and morale within university environments.

## Author contributions

All four authors, SP, AW, CP, and CB contributed to the formulation and writing of this paper. SP also undertook the analyses.

## Funding

The research reported in this paper was supported by grants from the Australian Research Council and the National Tertiary Education Union, and contributions from the Vice Chancellors of the participating universities. The first author was supported by a Bellberry PhD Scholarship.

### Conflict of interest statement

The authors declare that the research was conducted in the absence of any commercial or financial relationships that could be construed as a potential conflict of interest.
